# Supplementary material for: Asynchronous magnetic resonance elastography: Shear wave speed reconstruction using noise correlation of incoherent waves
Source: Magn Reson Med. 2022 Oct 27;89(3):990–1001. doi: 10.1002/mrm.29502 (PMC9792433; doi:10.1002/mrm.29502)
Supplement: Supplementary file 1 — DATA S1 MATLAB code used to generate the numerical simulations of Figure 1 [file MRM-89-990-s002.zip › k-Wave/helpfiles/makeCartSphere.html]

makeCartSphere :: Functions (k-Wave)


# makeCartSphere

Create a 3D Cartesian sphere.

## Syntax

```
sphere = makeCartSphere(radius, num_points)
sphere = makeCartSphere(radius, num_points, center_pos)
sphere = makeCartSphere(radius, num_points, center_pos, plot_sphere)
```

## Description

`makeCartSphere` creates a 3 x `num_points` array of the Cartesian coordinates of points evenly distributed over a sphere using the Golden Section Spiral method. The function is based on code by Patric Boucher and Henry Bland on http://www.xsi-blog.com. The 3D plot that is displayed after calling `makeCartSphere(10, 500, [0,0,0], true);` is given below.

## Inputs

|  |  |
| --- | --- |
| `radius` | sphere radius [m] |
| `num_points` | number of points on the sphere |

## Optional Inputs

|  |  |
| --- | --- |
| `center_pos` | [x, y, z] position of the circle center [m] (default = [0, 0, 0]) |
| `plot_sphere` | Boolean controlling whether the Cartesian coordinates are plotted using `plot3` (default = false) |

## Outputs

|  |  |
| --- | --- |
| `sphere` | 3 x `num_points` array matrix of Cartesian points |

## Examples

- 3D Time Reversal For A Spherical Sensor

## See Also

`cart2grid`, `makeCartCircle`, `makeSphere`
